# Somatic mutations in *ZFHX4* gene are associated with poor overall survival of Chinese esophageal squamous cell carcinoma patients

**DOI:** 10.1038/s41598-017-04221-7

**Published:** 2017-07-10

**Authors:** Tao Qing, Sibo Zhu, Chen Suo, Lei Zhang, Yuanting Zheng, Leming Shi

**Affiliations:** 1 Center for Pharmacogenomics, School of Pharmacy and Shanghai Cancer Center, Fudan University, Shanghai, China; 2 Collaborative Innovation Center of Genetics and Development, Fudan University, Shanghai, China

## Abstract

Recent genome-sequencing studies have revealed dozens of genes frequently mutated in esophageal squamous cell carcinoma, but few genes are associated with patients’ clinical outcomes. Novel prognostic biomarkers are urgently needed in the clinic. We collected both somatic mutations and clinical information of 442 Chinese esophageal squamous cell carcinoma patients from four published studies. Survival analysis was performed to reveal the clinical significance of the mutated genes. Dysregulation of the mutated genes was observed from public gene-expression data sets and its effects on cell migration and invasion were investigated with siRNA-mediated silencing. Our integrated analysis revealed 26 genes significantly and frequently mutated in esophageal squamous cell carcinoma. Importantly, mutations in *ZFHX4, SPHKAP, NRXN1*, *KIAA1109*, *DNAH5* and *KCNH7* were associated with poor survival. In addition, *ZFHX4* was overexpressed in tumor tissues compared to normal controls, and knockdown of *ZFHX4 in vitro* significantly inhibited cell migration and invasion. Mutations in *ZFHX4* were strongly associated with poor prognosis and the down-regulation of *ZFHX4* inhibits the progression of esophageal squamous cell carcinoma. Further investigation is warranted to confirm the prognostic values of *ZFHX4* in a prospective study.

## Introduction

Esophageal squamous cell carcinoma (ESCC) is one of the most common cancers in the world^1^. About 70% ESCCs occur in China, with the highest incidence rate in the Taihang Mountain region and Chaoshan district^2, 3^. ESCC is a rapidly progressing cancer with highly diverse clinical manifestations and outcomes, partially accounting for poor prognosis in late-stage patients. In the past decades, many environmental risk factors, such as smoking, drinking and hot food eating have been revealed to contribute to ESCC tumorigenesis^4, 5^. More recently, genome-wide association studies have identified dozens of high risk loci in ESCC, providing insights into the genetic basis of this deadly disease^6^. However, there remains a lack of effective prognostic biomarkers and therapeutic targets for ESCC.

High-throughput genome sequencing has revolutionized the field of cancer research. It can be utilized to characterize the alterations of an individual cancer genome, providing a comprehensive way to identify somatic mutations that might contribute to the initiation and progression of cancer. Recently, paired tumor/normal samples of Chinese ESCC patients have been sequenced in four independent studies^7–10^, with sample size varying from 88 to 139. Seventeen significantly mutated genes (SMGs), including *TP53, PIK3CA, NOTHC1, CDKN2A*, *NFE2L2* and *MLL2* mutated in at least 10% of ESCCs. These genome sequencing studies have provided a relatively comprehensive landscape of ESCC. However, due to the limited sample sizes and incomplete follow-up information, only a few mutated genes were shown to be associated with clinical outcomes. The roles of a large number of somatic mutations in ESCC still remain unknown.

The aim of this study is to provide a comprehensive view of mutational profiles and identify potential prognostic biomarkers associated with clinical outcomes of ESCC. Thus, we conducted a pooled analysis of the sequencing-based genotyping data from the aforementioned four genome-sequencing studies for a total of 442 Chinese ESCC patients^7–10^. Moreover, microarray gene-expression data of 17 additional ESCC patients^11^ were obtained from Gene Expression Omnibus (GEO) database^12^. We found that mutations in *ZFHX4, SPHKAP, NRXN1, KIAA1109, DNAH5* and *KCNH7* were associated with poor overall survival. We also examined somatic mutations and expression profiles of *ZFHX4* in The Cancer Genome Alas (TCGA) datasets and found that the mutations of *ZFHX4* were also associated with poor overall survival of liver hepatocellular carcinoma patients. Furthermore, expression of *ZFHX4* was widely dysregulated in many cancer types, indicating the significance of *ZFHX4* in cancer. *In vitro* siRNA-mediated silencing of *ZFHX4* effectively inhibited migration and invasion abilities in two ESCC cell lines.

To the best of our knowledge, this is the first study providing direct and strong evidence regarding the role of *ZFHX4* in cancer progression at both mutation and gene-expression levels. The significance of our study warrants further investigation into the potential prognostic and therapeutic utilities of *ZFHX4*.

## Materials and Methods

### Data description and processing

Metadata including sample size, sequencing information and the SMGs originally identified in the four previously published cancer genome studies were summarized^7–10^ (Table 1 **)**. The tumor and matched normal samples from 442 patients were sequenced with next-generation sequencing technologies, yielding 32,493 somatic mutations^7–10^. Among the 442 patients, 250 were collected by the Chinese Academy of Medical Sciences^8, 9^ and Linxian Cancer Hospital^9^, and the remaining 192 patients were from areas with high ESCC incidence rate in China^7, 10^, including 104 patients from Taihang Mountains region^10^ and 88 patients from Chaoshan District in Guangdong Province^7^. Linxian, Taihang Mountains region and Chaoshan District were three areas with the highest ESCC incidence rate in China^2, 13^. None of the patients received chemo/radiotherapy before sample collection, with surgery being their primary treatment. The survival time of 281 out of 442 patients was available. The survival status was indicated with “alive”, “deceased” and “loss” at the last follow-up.

**Table 1:** Summary of four cancer genome sequencing studies of ESCC in Chinese populations.

Ethnicity	SMGs	q value cut off	Number of patients	Number of patients with survival data	Tissue types	Protocols**	Average sequencing depth	Number of somatic mutations
**Lin** ***et al***. **(2014)**								
Chinese	*TP53, NOTCH1, RB1, NFE2L2, CDKN2A, FAT1, FAT2, ZNF750, PIK3CA, MLL2 (KMT2D), PTEN, EP300, KDM6A* (n = 13)	<0.2	139*	0	Tumor tissue/matched normal	WES/CETS	WES: 79×CETS: 111×	3,043
**Gao** ***et al***. **(2014)**								
Chinese	*TP53, NOTCH1, RB1, NFE2L2, CDKN2A, JUB (AJUBA)* (n = 6)	<0.1	113	113	Tumor tissue/matched normal	WES	WES: 122×	12,022
***et al***. **(2015)**								
Chinese	*TP53, NOTCH1, RB1, CDKN2A, ZNF750, PIK3CA, JUB (AJUBA), FBXW7, FAT1* (n = 9)	<0.1	104	80	Tumor tissue/matched normal	WGS/WES	WGS: 69×WES: 132×	10,149
**Song** ***et al***. **(2014)**								
Chinese	*TP53, CDKN2A, RB1, NFE2L2, ADAM29, FAM135B, PIK3CA, NOTCH1* (n = 8)	<0.1	88	88	Tumor/peripheral blood	WGS/WES	WGS: 38×WES: 126×	7,279

*Two patients without any gene mutated were excluded in the analysis.

**WES: whole-exome sequencing; WGS: whole-genome sequencing; CETS: coding exons targeted sequencing.

In order to use a consistent annotation across the four studies to facilitate pooled analysis, the annotation information of somatic mutations originally annotated with hg18 in the study of Gao *et al*.^9^ was converted to hg19 annotation using the UCSC LiftOver online tool (https://genome.ucsc.edu/cgi-bin/hgLiftOver). Moreover, the somatic mutations of *ZFHX4* in 12 cancer types were collected from the TCGA research network (http://cancergenome.nih.gov/), and the 12 cancer types were listed in Supplementary Table 1.

The gene-expression data of tumor and matched normal samples for 17 Chinese ESCC patients were available and downloaded from the Gene Expression Omnibus (GEO)^12^ dataset GSE20347^11^. Gene-expression profiles of the 17 pairs of samples were preprocessed by using Affymetrix Expression Console software, then normalized using Robust Multiarray Averaging (RMA) method^14^, followed by log2 transformation. In addition, RNA-seq data of tumor and matched normal samples of the 12 cancer types were downloaded from TCGA. The values of fragments per kilobase of transcript per million mapped reads (FPKM) of each sample were transformed to log2 scale. Differential expression analysis was performed by comparing the expression values between tumor and normal samples. For both microarray and RNA-seq data, a fold change > 1.5 and an adjusted p value < 0.05 from t-test were employed as the criteria for selecting genes differentially expressed between tumor and normal samples.

### Somatic mutations and identification of SMGs

In this study, 32,493 somatic mutations were identified from 442 ESCC patients. SMGs were analyzed with MutSigCV^15^ that detects genes associated with cancer as mutated more frequently than by chance. Default parameters were employed in this analysis. Genes with an FDR adjusted MutSigCV q-value < 0.2 were considered as SMGs.

### Survival analysis for mutated genes

First, we conducted survival analysis for genes mutated in at least 10 (out of 281) patients. Patients were divided into two groups according to the mutation status of each gene. The difference of overall survival between the two groups was estimated using Kaplan–Meier curve and tested for significance using log-rank test. Secondly, given that missense mutations and frame shift indels were dominant in ESCC and might alter protein structures and functions, we restricted the survival analysis on missense mutations and frame shift indels. A gene was considered to be associated with ESCC clinical outcomes when the p value of the log-rank test is <0.05. For the TCGA datasets, survival analysis was performed according to the mutation status of *ZFHX4* in patients in each cancer type. False discovery rate was calculated from p values with multiple-comparison correction. The log-rank test and Kaplan–Meier methods were performed using the “survival” package in R^16^.

### Culture of ESCC cell lines

The human esophageal cancer cell lines KYSE150 and TE-1 were maintained in RPMI 1640 medium with 10% fetal bovine serum (FBS), 2 mM L-glutamine, 2500 IU/mL penicillin and 5 mg/mL streptomycin (all from Sigma). The cells were maintained at 37°C in a humidified atmosphere of 5% CO_2_. We confirmed the identities of these cell lines by matching the short tandem repeat (STR) profiles to the DSMZ online STR database^17^.

### Quantitative real-time PCR

Real-time PCR was performed to examine the gene-expression levels of *ZFHX4* and *GAPDH* in KYSE150 and TE-1 cell lines using the Premix Ex Taq kit (Takara) and a 7300 real-time PCR system (Life Technologies) according to the manufacturers’ instructions. Primers were designed as described below: ZFHX4 forward, 5′-GGAGAACTGTGGGCAGAGAG-3′; ZFHX4 reverse, 5′- AGGTAAGGTCCGCTTTGGTT-3′; GAPDH forward, 5′-TCTCTGCTCCTCCTGTTC-3′, and GAPDH reverse, 5′-GTTGACTCCGACCTTCAC-3′. The mRNA expression level of *ZFHX4* was normalized to that of *GAPDH*.

### siRNA knockdown of *ZFHX4* expression

For siRNA treatment, cells were plated in 24-well plates at 2 × 10^5^ cells/well 24 h before transfection. Cells were transfected with siRNA using Lipofectamine 2000 (Invitrogen, Carlsbad, California, USA) and Opti-MEM (Invitrogen) according to the manufacturers’ instructions. Final siRNA concentrations were 10 nmol/L in experiments using *ZFHX4*-targeting siRNA molecules. The plates were returned to the incubator until migration assays or wound healing assays were carried out. siRNA molecule (GenePharma, China) sequences *ZFHX4*-siRNA 5′GCAGGUCUCGAGGAUUCAATT and 5′UUGAAUCCUCGAGACCUGCTT were used to knock down *ZFHX4* expression. The control siRNA GGACGCAUCCUUCUUAA was used as a nonfunctional siRNA control. To monitor the knockdown efficiency, cell lysates were harvested for qPCR quantification of *ZFHX4* expression.

### *In vitro* cell motility invasion assay

Motility invasion assays were carried out in transwell dishes (Corning, USA), with 8 uM pores. After 6 h of transfection with siRNA or control RNA, cells were harvested and plated in complete medium on top of the culture insert at 2 × 10^5^ cells/insert in 0.5 mL. The lower chamber contained 0.75 mL complete medium. Inserts were incubated at 37°C and 5% CO_2_ for 48 h. Non-invading cells were removed with a cotton swab soaked in medium. Cells that had moved through the pores (to the lower surface of the filters) were incubated with CCK-8 viability assay reagents (Dojindo, Japan) for 3 h and OD was measured at 450 nm with a spectrophotometer. Three inserts were counted for at least three times for each experiment.

### *In vitro* wound-healing and migration assays

KYSE150 and TE-1 cells were treated with siRNA as described above. Cells were incubated overnight yielding confluent monolayers for wounding that were made using a pipette tip and photographs taken immediately (time zero) and 24 h or 48 h after wounding for KYSE150 and TE-1 cells, respectively. The distance migrated by the cell monolayer to close the wounded area during this time period was measured. Results were expressed as a migration index, that is, the distance migrated by siRNA treated (control or targeted) relative to the distance migrated by non-targeting siRNA treated cells. Experiments were carried out in triplicates and measured at least five times per assay.

## Results

### Pooled analysis identify novel SMGs of ESCC

Previous studies^7–10^ reported a total of 17 genes significantly mutated in Chinese ESCC patients, including *TP53*, *CDKN2A*, *FBXW7*, *PIK3CA* and *NFE2L2*. The detailed information about the SMGs, sample size, sequencing protocol and clinical information was summarized in Table 1. To conduct a comprehensive analysis of the somatic mutation profiles, we integrated the 32,493 somatic mutations from the 442 ESCC patients, with an average of 74 mutations per patient. Sixty-six percent (66%) of the somatic mutations are missense and indels, and were detected in 239 (54%) patients. The 32,493 somatic mutations were located in 12,074 human genes, including 407 genes recorded in the Catalogue of Somatic Mutations in Cancer database (COSMIC)^18^. Forty-five (45) genes showed a mutation frequency of over 5% (Supplementary Fig. 1). Not surprisingly, *TP53* mutations are the most common genetic events occurring in 79.4% patients, followed by nine genes mutated in more than 10% of the patients, including *TNN* (36.4%), *MUC16* (16.3%), *MLL2* (14.3%), *CSMD3* (13.6%), *NOTCH1* (12.6%), *FAT1* (12.2%), *PCLO* (12.2%), *SYNE1* (12.0%) and *LRP1B* (11.3%).

**Figure 1:**
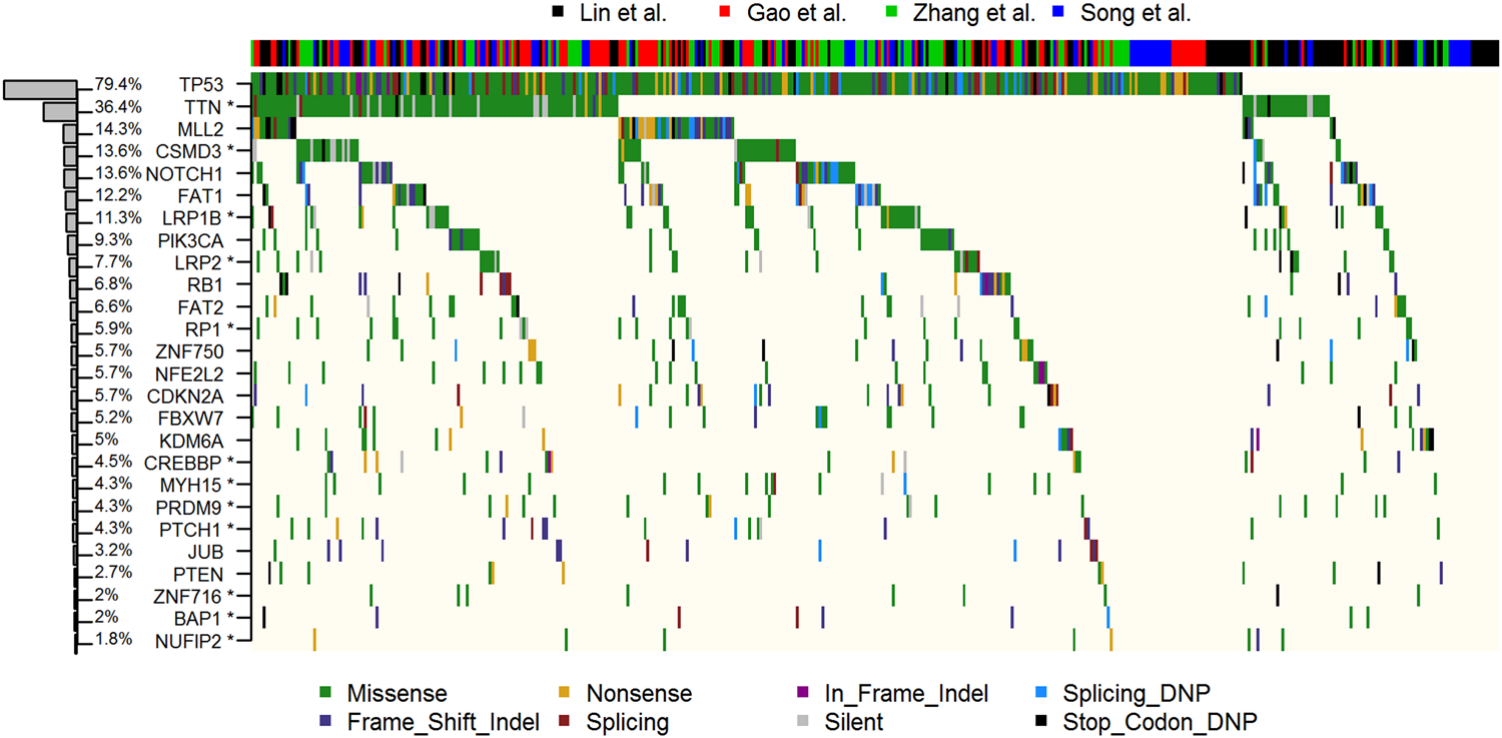
Landscape of the significantly mutated genes in ESCC. Top: four datasets. Left: SMGs were sorted according to mutation frequency in 442 patients, total number of somatic mutations of each gene, and the percentages indicated the fraction of patients with the individual mutation. Genes marked with an asterisk have a q value of <0.2 in the MutSigCV analysis. Middle: mutations in 26 frequently mutated genes across 442 ESCC samples. Each column represents an individual, each row represents a gene, and mutation subtypes are denoted by color.

The large sample size of 442 patients could have empowered the detection of more genes associated with ESCC, significantly mutated gene analysis was performed with the pooled 32,493 somatic mutations identified from the 442 ESCC patients by using MutSigCV^15^. As a result shown in Fig. 1, 12 novel SMGs (q < 0.2) were detected including TTN, CSMD3, LRP1B, LRP2, RP1, CREBBP, MYH15, PRDM9, PTCH1, ZNF716, BAP1 and NUFIP2. Fourteen previously reported SMGs were identified in the pooled analysis including *CDKN2A*, *FBXW7*, *JUB*, *NFE2L2*, *NOTCH1*, *PIK3CA*, *PTEN*, *MLL2*, *TP53*, *FAT1*, *ZNF750*, *RB1*, *KDM6A* and *FAT2*. In addition, *CDKN2A*, *FBXW7*, *JUB*, *NFE2L2*, *NOTCH1*, *PIK3CA*, *PTEN*, *MLL2* and *TP53* were ranked as the top SMGs. The results of SMGs were listed in Supplementary Table 2.

### Survival analysis identified novel prognostic markers of ESCC

Survival analysis can be used to identify associations between genetic variations and clinical outcomes of interest. In this study, we pooled the genomic and survival information from the four studies together and found that 281 patients with survival information can be used for assessing the clinical significance of the mutated genes. The median follow-up time was 2.5 years, and 231 (82%) patients had been followed up for more than one year since diagnosis.

**Figure 2:**
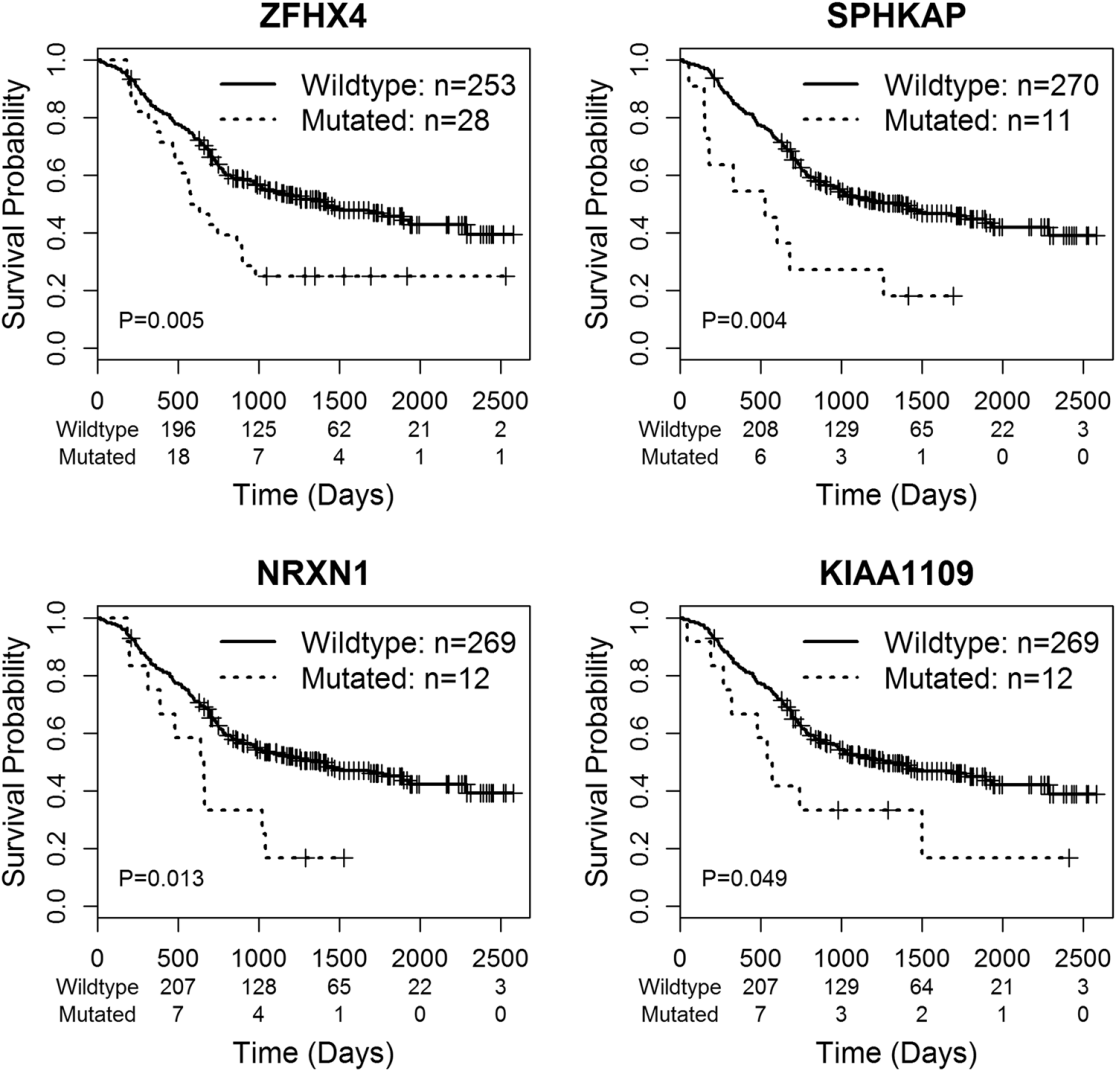
Survival analysis for mutations of frequently mutated genes. The overall survival rates of patients according to gene mutation status were shown. Wildtype indicates a group of patients without gene mutation. Mutated means that patients carry gene mutation. P values were calculated by the log-rank test.

**Figure 3:**
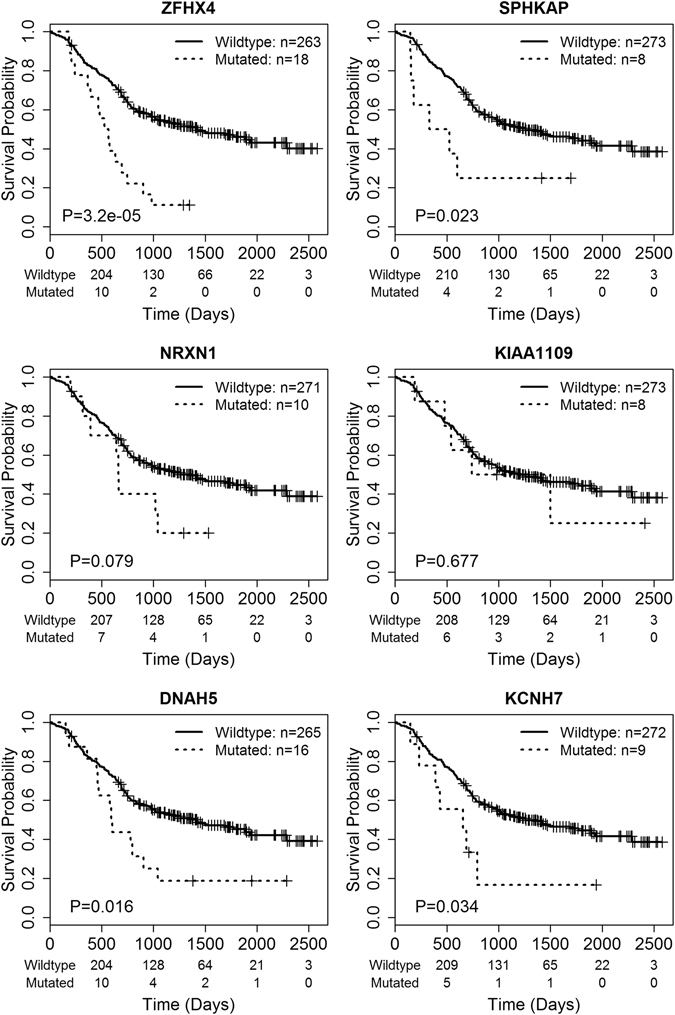
Survival analysis for missense mutations and frame-shift indels of frequently mutated genes. The overall survival rates of patients according to gene mutation status were shown. Only missenses mutations and frame shift indels were considered for each gene. Wildtype indicates a group of patients without a gene mutation. Mutated means that patients carry a gene mutation. P values were calculated by the log-rank test.

Kaplan–Meier analysis showed that mutations in *ZFHX4*, *SPHKAP*, *NRXN1* and *KIAA1109* were significantly (p < 0.05) correlated with poor overall survival (Fig. 2). Notably, *ZFHX4* mutations were detected in 8.6% (38/442) of the patients. Missense mutations and frame-shift indels were the most deleterious mutation types in cancer, because they change gene-coding sequences, potentially altering protein functions. We performed survival analysis for missense mutations and frame-shift indels alone for each gene. Overall survival of patients with *ZFHX4* and *SPHKAP* mutations (missense and frame shift) decreased dramatically (log-rank test p value = 3.2 × 10^−5^ and 0.023, respectively, Fig. 3). In addition, we identified two additional genes *DNAH5* and *KCNH7*, whose missense mutations and frame-shift indels are strongly associated with patient survival (a log-rank test p-value of 0.016 and 0.034, respectively). However, the missense and frame shift variations of *NRXN1* and *KIAA1109* were not associated with clinical outcomes (Fig. 3).

**Figure 4:**
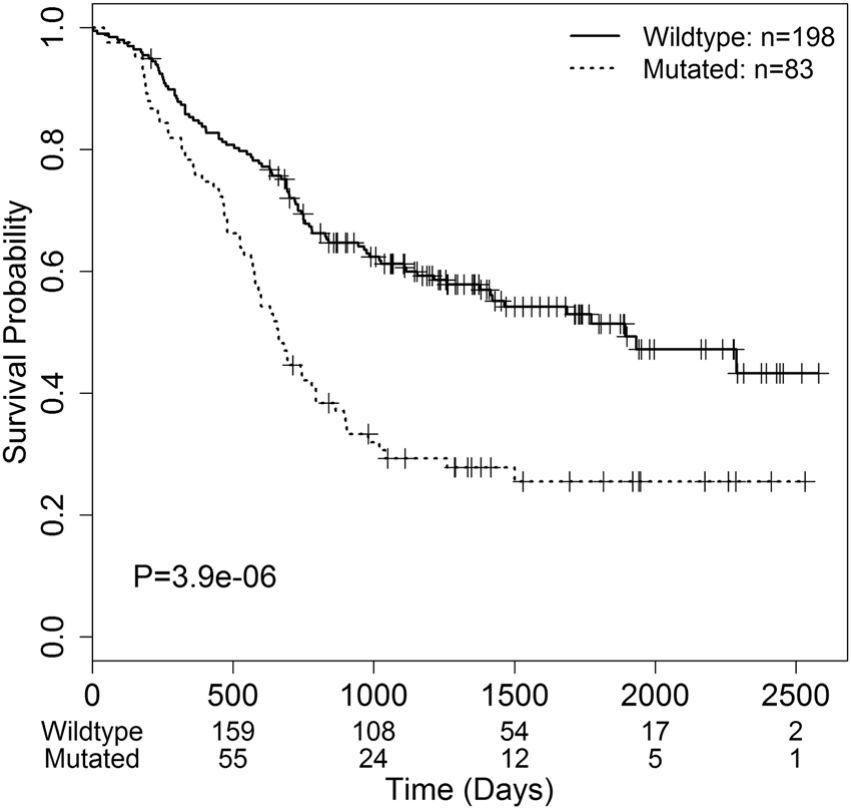
Survival analysis for the combination of all the mutations identified in six genes (*ZFHX4*, *SPHKAP*, *NRXN1*, *KIAA1109*, *DNAH5* and *KCNH7*). The overall survival rates of patients according to gene mutation status were shown. Wildtype indicates a group of patients without a single mutation in any of the six genes. Mutated means that patients carry a gene mutation. P value was calculated by the log-rank test.

To further confirm the combined predictability of the survival-associated genes, we investigated the effect on survival of the mutation status of any of the six genes, i.e., *ZFHX4*, *SPHKAP*, *NRXN1*, *KIAA1109*, *DNAH5* and *KCNH7*. That is, if a mutation happens in any of the six genes in a patient, then the patient is considered to be a mutation carrier. As shown in Fig. 4, 83 patients who carried at least one mutation in at least one of the six genes exhibited significantly poorer survival with a log-rank test p-value of 3.9 × 10^−6^, suggesting that integrating mutations of multiple genes could better predict patient overall survival.

### *ZFHX4* mutations in TCGA dataset

As *ZFHX4* mutations significantly contributed to poor survival of Chinese ESCC patients, we further examined the prognostic role of *ZFHX4* mutations in 12 cancer types investigated by TCGA including ESCC and esophageal adenocarcinoma (EAD) of Vietnam, Brazil, United States and other populations^19^. *ZFHX4* mutations were widely detected in 12 cancer types as listed in Supplementary Table1. The mutation rate of *ZFHX4* in 12 cancer types ranged from 2.2% (prostate adenocarcinoma, PRAD)^20^ to 43.8% (lung adenocarcinoma, LUAD)^21^. The mutation map in Supplementary Fig. 2A showed the distribution of somatic mutations on the gene body of *ZFHX4* and several recurrent mutations in cancer were observed, such as p.L408fs/G407fs and p.P1042P/S. As shown in Supplementary Fig. 2B, mutations of *ZFHX4* were correlated with poor overall survival of liver hepatocellular carcinoma (LIHC) patients with a log-rank test p-value of 0.01. However, although *ZFHX4* mutations were observed in eight ESCC patients in the TCGA datasets, there was no correlation between *ZFHX4* mutations and survival of ESCC patients, EAD (Supplementary Fig. 2C) and the remaining cancer types (Supplementary Table 1).

### *ZFHX4* expression in cancer

To further demonstrate the important role of *ZFHX4* gene in cancer, we examined the gene-expression profile of *ZFHX4* in Chinese ESCC and 12 cancer types in TCGA. The expression of *ZFHX4* was widely altered in tumor tissues. As shown in Supplementary Fig. 3, expression of *ZFHX4* was significantly up-regulated in Chinese ESCC^11^, LUAD and LUSC^21, 22^, and was down-regulated in BRCA, LIHC, rectum adenocarcinoma (READ) and stomach adenocarcinoma (STAD)^23–25^. Moreover, the expression value of tumor samples was 1.6 times higher than the normal samples of ESCC in TCGA dataset^19^, exactly the same as observed in the 17 ESCC patients, but the p value (0.3) was not significant due to very small sample size (3).

### Expression of *ZFHX4* was associated with migration and invasion ability of ESCC

**Figure 5:**
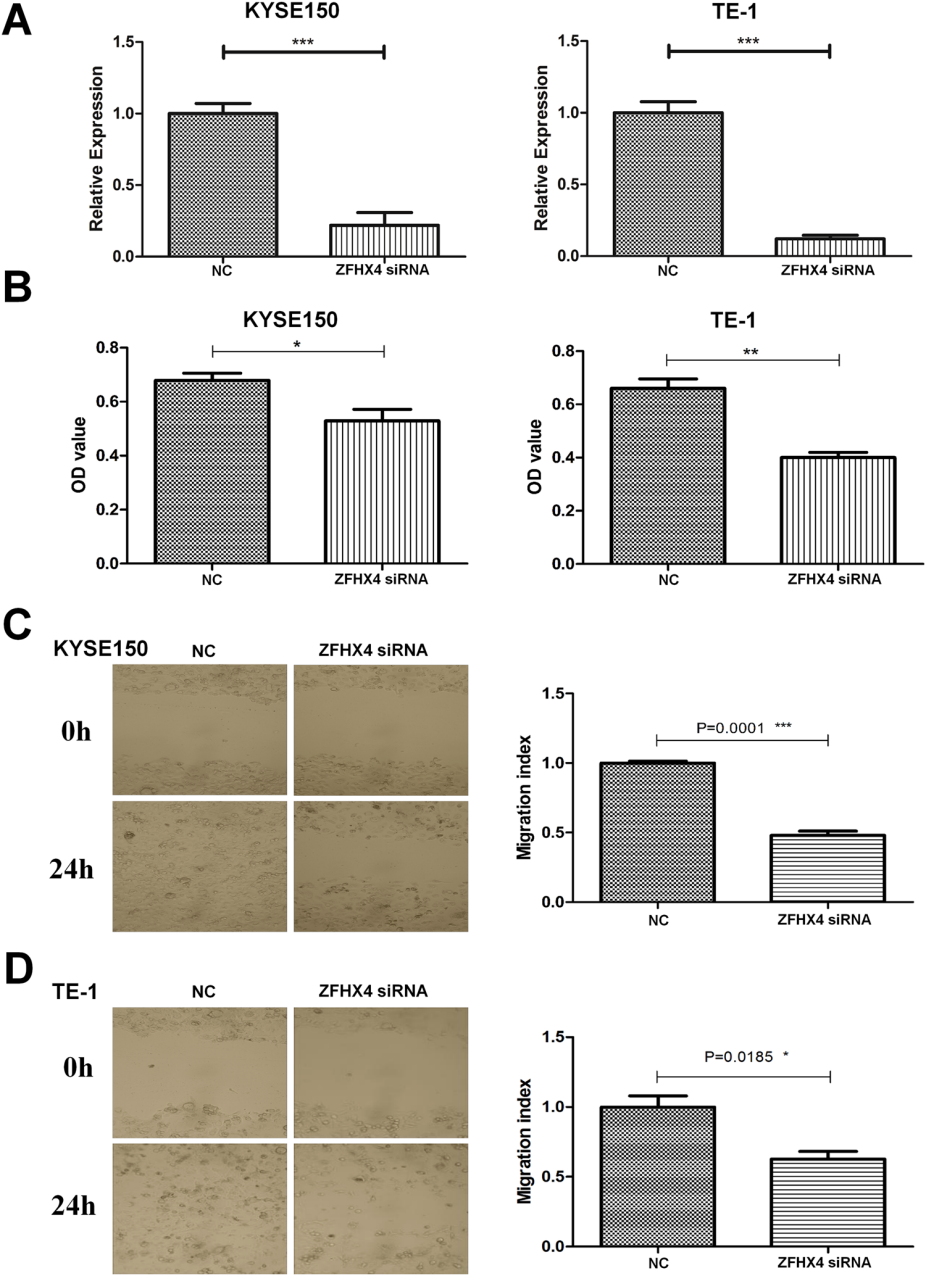
Validation of the biological functions of *ZFHX4* with RNA interference. Data and images were shown for two cell lines: KYSE150 and TE-1. (**A**) After 48 h, *ZFHX4* expression was evaluated by real-time RT–PCR in control (NC) and siRNA of ZFHX4. (**B**) *In vitro* invasion activity of KYSE150 and TE-1 cells after treatments with siRNA of *ZFHX4*. (**C**,**D**) The images showed distance migrated by the cell monolayer to close the wounded area at starting point and after 24 h of ZFHX4 knockdown in KYSE150 and TE-1 cell lines. Bars represent the motility index of each treatment, and all data represented the mean ± s.d. of three replicates. The p values were calculated using the Student’s paired t-test, two-tailed. *p < 0.05; **p < 0.01.

Given the facts that mutations in *ZFHX4* were frequently observed in ESCC patients and were associated with poorer survival and that *ZFHX4* was overexpressed in ESCC, we knocked down *ZFHX4* expression in KYSE150 and TE-1 cell lines using RNA interference sequences to determine whether *ZFHX4* plays an important role in ESCC progression. We first checked the expression level of *ZFHX4* in KYSE150 and TE-1 cell lines. The mean delta Ct values between *GAPDH* and *ZFHX4* were 13.5 and 16.1 in the KYSE150 and TE-1 cells, respectively. In Fig. 5A, real time reverse transcription PCR (RT–PCR) assays showed that the mRNA expression of *ZFHX4* was reduced by 78.1% in KYSE150 and 88.0% in TE1 due to siRNA silencing.

Transwell motility assays were used to estimate the effect of *ZFHX4* gene knockdown on invasion ability. ESCC cell lines treated with non-targeting siRNA were used as a control. The results of transwell invasion assays were shown in Fig. 5B. The invasion ability of KYSE150 cells with *ZFHX4* gene knockdown is impaired by 15.1% compared with their control cells. For TE-1, consistent with what was observed in KYSE150 cells, knockdown of *ZFHX4* expression inhibited cell invasion ability by 26.0%.

To further confirm the association of *ZFHX4* expression with ESCC, we performed an *in vitro* wound-healing assay to assess the effect of siRNA knockdown on cell motility. The migration index was estimated as an indication of the distance migrated by cells to close the wound after knockdown. As shown in Fig. 5C, compared to the non-targeting control group, the *ZFHX4* knockdown KYSE150 cells migrated with limited distances, and were unable to achieve wound closure after 24 h. Similarly, migration of *ZFHX4* knockdown TE-1 cells was significantly decreased (Fig. 5D). We also performed cell proliferation and apoptosis assays, but knockdown of *ZFHX4* showed no significant effects on cell proliferation and apoptosis (results not shown). In summary, these experimental results indicated that loss of *ZFHX4* resulted in impaired migration and invasion abilities of ESCC cells.

## Discussion

We aim to provide a comprehensive view of the somatic mutation landscape and reveal novel prognostic biomarkers by a pooled analysis of genome-sequencing data of 442 Chinese ESCC patients. First, the clinical significance of the mutated genes was explored. Survival analysis of frequently mutated genes in patients suggested that mutations in *ZFHX4*, *SPHKAP*, *NRXN1*, *KIAA1109*, *DNAH5* and *KCNH7* were associated with poor prognosis. Secondly, SMG analysis found that 26 genes were significantly mutated in ESCC, and 12 genes had not been reported as SMGs in previous ESCC studies. Finally, we observed that *ZFHX4* is overexpressed in ESCC. Knockdown of *ZFHX4* in two ESCC cell lines significantly inhibited the ability of cell migration and invasion. Importantly, to the best of our knowledge, our work represents the first study to show the potential prognostic value of *ZFHX4* in ESCC.

In this study, we validated the pathological functions of *ZFHX4* in ESCC cell lines. *ZFHX4* was evidently associated with ESCC in terms of mutation, gene-expression level and patient overall survival. Knockdown of *ZFHX4* expression significantly inhibited the migration and invasion of ESCC cells. *ZFHX4*, a member of the zinc finger family, was firstly identified in 1995^26^. However, the role of *ZFHX4* in cancer has not been well studied, especially in ESCC. One study reported that *ZFHX4* expression is required in differentiating P19 embryonal carcinoma cells and C2C12 myoblasts, highlighting the important roles of *ZFHX4* in neural and muscle development^27^. *ZFHX4* is a master regulator of *CHD4* and *SOX2*, and regulates the glioblastoma tumor initiating cell state^28^. *ZFHX4* silencing resulted in decreased tumorigenesis and prolonged cancer-free survival of glioblastoma^28^. Our data provided strong evidence that mutations in *ZFHX4* contribute to the development and progression of ESCC.

We further examined the prognostic role of *ZFHX4* mutations in 12 cancer types in TCGA. Mutations of *ZFHX4* significantly contributed to the poor survival of LIHC patients (p = 0.012) as shown in Supplementary Fig. 2B. However, mutations of *ZFHX4* were not significantly associated with patient survival of both ESCC and EAD in TCGA^19^ (Supplementary Fig. 2C). Several reasons might account for the apparently inconsistent results between different ESCC datasets. First, ESCC patients of TCGA were from Vietnam (n = 41; 45.5%), Brazil (n = 15; 16.7%), the United States (n = 14; 15.5%), Russia (n = 12; 13.3%), Ukraine (n = 4; 4.4%) and Canada (n = 4; 4.4%)^19^. Different populations of diverse ethnic backgrounds may have different risk factors and mechanisms of tumor initiation and progression, limiting the power of survival analysis. Secondly, only 8 of the 91 patients in the TCGA dataset carried *ZFHX4* mutations^19^. Finally, the median follow-up time of the ESCC patients in the TCGA study was 1.0 year that was much shorter than the median survival time of 2.5 years of Chinese patients^7, 9, 10, 19^. The small sample size and short survival time might not be enough to make a confident survival analysis for the TCGA dataset.

*ZFHX4* expression was widely dysregulated in cancer. *ZFHX4* was significantly up-regulated in tumor tissues of Chinese ESCC, LUAD and LUSC^21, 22, 29^, but it was not significantly altered in ESCC of TCGA which might be because of the much smaller sample size (n = 3)^19^. We observed the down-regulation of *ZFHX4* in several cancer types, such as LIHC, BRCA and READ^23, 24^. The inconsistency in *ZFHX4* regulation between different cancer types may indicate the complex mechanism of *ZFHX4* in tumor biology. As a new candidate gene of cancer, the functionality of *ZFHX4* is not well studied. Thus, more research is needed to further reveal the important roles of *ZFHX4* in the progression of cancer.

By pooling genome-sequencing data and clinical information of four previously published studies with a much larger sample size of 442 patients, we were able to detect additional ESCC-related genes. In our analysis of SMGs, we discovered 12 additional SMGs of ESCC that have not been identified in the four original individual studies^7–10^, where the sample size varied from 88 to 139. Genes with low mutation frequency across samples, but with functional implications might be overlooked when the sample size is small. Recent studies have reported that some of the 12 additional genes, such as *CSMD3* and *LR1B* ^30, 31^, indeed are associated with ESCC progression, indicating the power and validity of our pooled analysis.

In summary, our study represents the most comprehensive survey of the mutational landscape of ESCC in Chinese populations. The largest integrative genomic dataset facilitated the identification of additional significantly mutated genes in ESCC. In addition, our analysis allowed for the discovery of novel mutation biomarkers for predicting the prognosis of ESCC patients. The clinical utilities of the newly identified prognostic biomarkers, especially *ZFHX4*, warrant further validation in new patient cohorts.

## Electronic supplementary material

Supplementary Information
