# Supplementary material for: Somatic mutations in ZFHX4 gene are associated with poor overall survival of Chinese esophageal squamous cell carcinoma patients
Source: Sci Rep. 2017 Jul 10;7:4951. doi: 10.1038/s41598-017-04221-7 (PMC5504002; doi:10.1038/s41598-017-04221-7)
Supplement: Supplementary file 1 — Supplementary Information [file 41598_2017_4221_MOESM1_ESM.pdf]

Supplementary Information

Somatic mutations in *ZFHX4* gene are associated with poor overall survival of Chinese esophageal squamous cell carcinoma patients

Tao Qing[1†], Sibo Zhu[2†], Chen Suo[2], Lei Zhang[2], Yuanting Zheng[1,2*], Leming Shi[1,2*]

[1] Center for Pharmacogenomics, School of Pharmacy and Shanghai Cancer Center, Fudan University, Shanghai, China

[2] Collaborative Innovation Center of Genetics and Development, Fudan University, Shanghai, China

[†] Equal contributors

[*]To whom correspondence should be addressed to Yuanting Zheng (yuantingzheng@fudan.edu.cn) or Leming Shi (lemingshi@fudan.edu.cn).

Supplementary Table 1. *ZFHX4* mutations in 12 types of cancer studied by TCGA.

	Cancer	Number of Samples	Number of Samples with Mutations	Mutation Frequency (%)	Log-rank P Value	Adjusted P Value (FDR)
1	Liver hepatocellular carcinoma (LIHC, unpublished)	373	24	6.4	0.01	0.12
2	Colon adenocarcinoma (COAD)[1]	217	57	26.3	0.16	0.71
3	Lung adenocarcinoma (LUAD)[2]	543	184	33.9	0.22	0.71
4	Head and neck squamous cell carcinoma (HNSC)[3]	512	57	11.1	0.33	0.71
5	Esophageal adenocarcinoma (EAD)[4]	79	12	15.2	0.39	0.71
6	Breast cancer (BRCA)[5]	982	38	3.9	0.41	0.71
7	Stomach adenocarcinoma (STAD)[6]	379	63	16.6	0.44	0.71
8	Prostate adenocarcinoma (PRAD)[7]	499	11	2.2	0.47	0.71
9	Lung squamous cell carcinoma (LUSC)[8]	178	78	43.8	0.75	0.91
10	Pancreatic ductal adenocarcinoma (PAAD, unpublished)	185	7	3.8	0.76	0.91
11	Rectal adenocarcinoma (READ)[1]	81	11	13.6	0.96	0.96
12	Esophageal squamous cell carcinoma (ESCC)[4]	91	8	8.8	0.96	0.96

Supplementary Table 2. Results of MutSigCV analysis (q < 1).

	gene	N_noncoding	n_nonsilent	n_silent	n_noncoding	p	q
1	CDKN2A	0	25	0	0	0	0
2	FBXW7	0	24	1	0	0	0
3	JUB	0	14	0	0	0	0
4	NFE2L2	0	25	0	0	0	0
5	NOTCH1	0	58	3	0	0	0
6	PIK3CA	0	42	0	0	0	0
7	PTEN	0	12	0	0	0	0
8	MLL2	0	67	1	0	2.22E-16	5.24E-13
9	TP53	0	387	4	0	4.44E-16	9.31E-13
10	FAT1	0	50	7	0	2.89E-15	5.14E-12
11	ZNF750	0	28	0	0	3.00E-15	5.14E-12
12	RB1	0	31	0	0	3.66E-15	5.76E-12
13	KDM6A	0	24	1	0	4.73E-12	6.86E-09
14	PTCH1	0	18	1	0	1.76E-09	2.38E-06
15	CREBBP	0	18	2	0	1.07E-07	1.35E-04
16	BAP1	0	9	0	0	1.58E-07	1.87E-04
17	FAT2	0	31	6	0	1.81E-05	2.01E-02
18	TTN	0	194	32	0	2.43E-05	2.54E-02
19	CSMD3	0	60	10	0	2.95E-05	2.93E-02
20	RP1	0	25	3	0	3.78E-05	3.56E-02
21	PRDM9	0	18	1	0	1.34E-04	1.20E-01
22	ZNF716	0	9	0	0	1.60E-04	1.37E-01
23	MYH15	0	18	1	0	1.91E-04	1.57E-01
24	NUFIP2	0	8	0	0	2.09E-04	1.63E-01
25	LRP2	0	35	4	0	2.16E-04	1.63E-01
26	LRP1B	0	46	7	0	2.29E-04	1.66E-01
27	CHEK2	0	8	1	0	3.94E-04	2.75E-01
28	ZFHX4	0	30	9	0	4.13E-04	2.78E-01
29	MAGEC1	0	15	2	0	5.07E-04	3.30E-01
30	C16orf3	0	4	0	0	5.37E-04	3.38E-01
31	GPR22	0	7	0	0	6.50E-04	3.87E-01
32	SLCO5A1	0	14	3	0	6.56E-04	3.87E-01
33	EP300	0	38	2	0	7.03E-04	4.02E-01
34	C5orf62	0	3	0	0	7.42E-04	4.11E-01
35	STAT4	0	6	0	0	8.27E-04	4.46E-01
36	DMD	0	36	3	0	1.10E-03	5.77E-01
37	SUFU	0	4	0	0	1.81E-03	9.21E-01

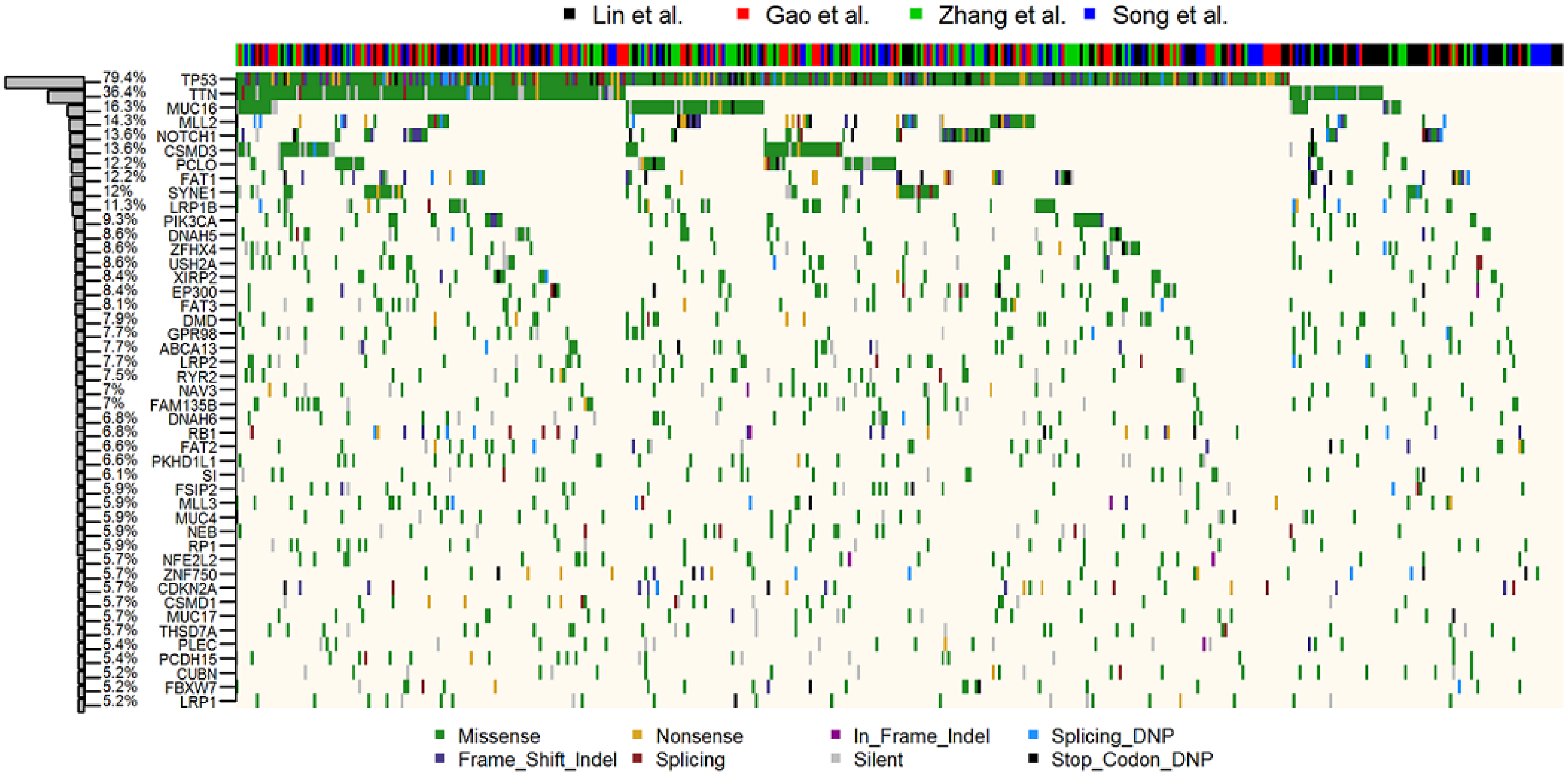

Supplementary Figure 1. Mutation map of 45 frequently mutated genes. Top: four datasets. Left: genes were sorted according to mutation frequency, total number of somatic mutations of each gene, and the percentages indicated the fraction of mutated individuals. Middle: mutations in frequently mutated genes across 442 ESCC samples. Each column represents an individual, each row represents a gene, and mutation subtypes are denoted by color.

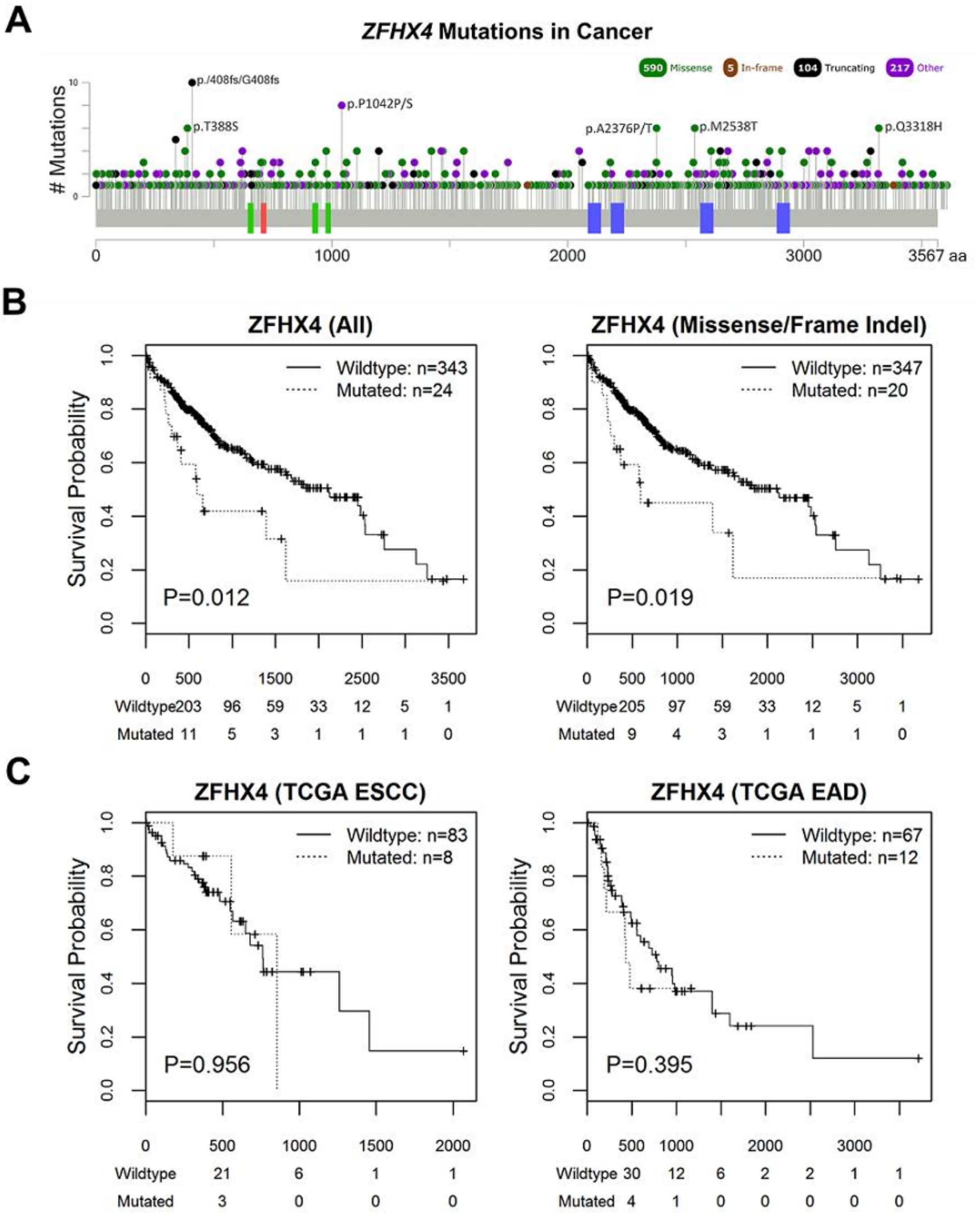

Supplementary Figure 2. *ZFHX4* mutations in cancer. (A) Mutation map of *ZFHX4* in Chinese ESCC and 12 TCGA cancer types. The x-axis indicates gene body of *ZFHX4* in amino acid function view. The y-axis indicates number of patients carrying *ZFHX4* mutations. The color of dots indicates mutation types of missense (green), in-frame indels (brown), truncation (black) and other mutation types (purple). (B) Survival analysis for *ZFHX4* mutations in LIHC of TCGA. The overall survival rates of patients according to gene mutation status were shown. Wildtype indicates a group of patients without gene mutation. Mutated means patients carrying gene mutations. P values were calculated by the log-rank test. (C) Survival analysis for *ZFHX4* mutations in ESCC and EAD of TCGA.

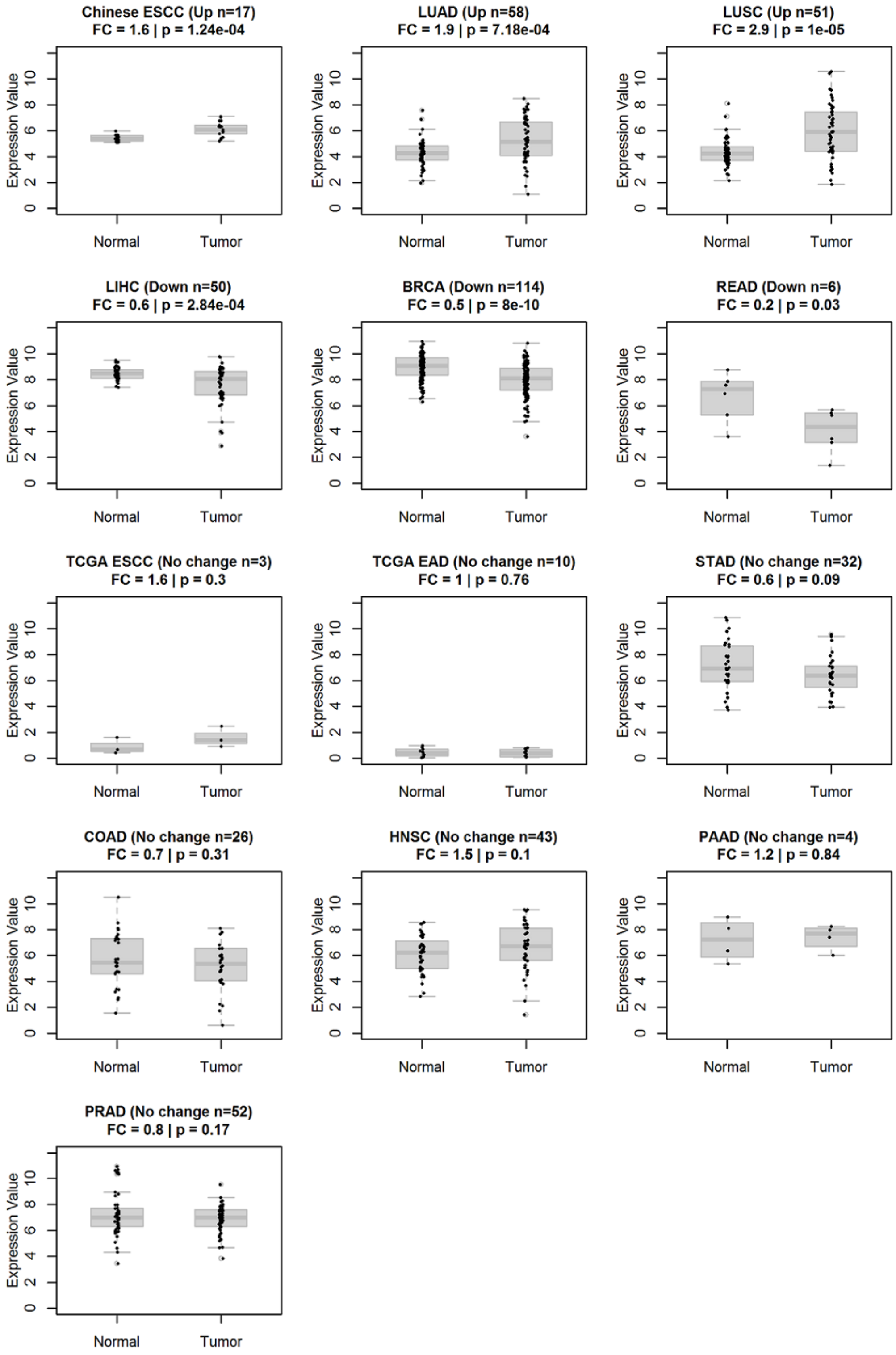

Supplementary Figure 3. Gene expression of *ZFHX4* in Chinese ESCC and 12 TCGA cancer types. Up-regulation, down-regulation and nonsignificant changes of expression between tumor and normal samples were marked by UP, Down and No change, respectively. n is the number of sample pairs. FC means fold change and p represents t-test p value.

References

1 Muzny, D. *et al.*, Comprehensive molecular characterization of human colon and rectal cancer. *Nature* **487** 330 (2012).

2 Collisson, E. *et al.*, Comprehensive molecular profiling of lung adenocarcinoma. *Nature* **511** 543 (2014).

3 Lawrence, M. *et al.*, Comprehensive genomic characterization of head and neck squamous cell carcinomas. *Nature* **517** 576 (2015).

4 Kim, J. *et al.*, Integrated genomic characterization of oesophageal carcinoma. *Nature* **541** 169 (2017).

5 Koboldt, D. *et al.*, Comprehensive molecular portraits of human breast tumours. *Nature* **490** 61 (2012).

6 Bass, A. *et al.*, Comprehensive molecular characterization of gastric adenocarcinoma. *Nature* **513** 202 (2014).

7 Abeshouse, A. *et al.*, The Molecular Taxonomy of Primary Prostate Cancer. *Cell* **163** 1011 (2015).

8 Hammerman, P. *et al.*, Comprehensive genomic characterization of squamous cell lung cancers. *Nature* **489** 519 (2012).
